# Comorbid anxiety has schizophrenia diagnosis and management modality

**DOI:** 10.1192/j.eurpsy.2023.1421

**Published:** 2023-07-19

**Authors:** A. Korchi, F. Laboudi, A. Ouanass

**Affiliations:** Hopitale psychiatrique, Kenitra, Morocco

## Abstract

**Introduction:**

Schizophrenia is a chronic psychiatric disorder with disorganization progressive and important affecting several spheres: thought, affectivity, cognition and social life of the subject. It is a serious mental disorder with a prevalence of nearly 1%.

Men would present more premorbid difficulties, but the overall prevalence of symptoms of schizophrenia is similar in both sexes.

When it comes to comorbidity, men and women are vulnerable to somewhat different health issues.

Anxiety symptoms are one of the main symptoms in patients with schizophrenia, but the effect of anxiety symptoms on patients is easily overlooked.

About 40% have persistent anxiety symptoms in patients with schizophrenia, and whether they are accompanied depressive symptoms, the symptoms may have a greater impact on the patients. We need to help patients avoid suicide when the symptoms depression appear.

Hallucinations auditory, anxiety symptoms and depressive symptoms may exist simultaneously in patients with schizophrenia.

Most studies focus on studying the relationship between auditory hallucinatory symptoms and depressive symptoms in patients with schizophrenia, the relationship between auditory hallucinatory symptoms and anxiety symptoms, or the relationship between anxiety symptoms and symptoms depressive

**Objectives:**

The objective of this study is to resolve important questions concerning the interaction of anxiety and schizophrenia in patients followed in psychiatry at Arrazi Hospital .

**Methods:**

Descriptive and analytical cross-sectional study, conducted over a period from May 2022 to October 2022 in patients consulted in Arrazi de Salé, using a questionnaire grouping together the sociodemographic characteristics, the risks and the advantages of antipsychotics, medical comorbidities and the mobilization of psychosocial support, and thus the BPRS Anxiety Rating Scale.

**Results:**

Of the 42 patients who completed the study, 18 were female and 24 were male 60% were single. The average age was 36.63 years, 60% have secondary education, 25% primary, and 15% university level, 30% have a family history of schizophrenia.

32 were taking an atypical antipsychotic, 10 were taking classic neuroleptics.

Most schizophrenics encountered in psychiatry are stabilized on antipsychotic treatment, and those who have an anxious comorbidity are still too often underestimated , put on the account of the positive symptoms and the negative symptoms of schizophrenia, it is insufficiently diagnosed and treated.

The effect of anxiety symptoms on patients is easily overlooked.

It should be kept in mind that anxiety in schizophrenia requires special attention when discussing and prescribing antipsychotic medications.

**Conclusions:**

Optimal interventions for patients with comorbid schizophrenia and anxiety differ by quality of life.

At all consultations, preventive strategies should consider mindful interviews and the risks and benefits of treatment for schizophrenia and comorbidities.

**Disclosure of Interest:**

None Declared

